# Correlates of Purpose in Life and Their Potential Role in Successful Aging Among Older Adults in Rural Japan

**DOI:** 10.3390/ejihpe15120250

**Published:** 2025-12-09

**Authors:** Haruka Ariji, Ishtiaq Ahmad, Yoshihisa Shirayama, Miyoko Okamoto, Hira Taimur, Motoyuki Yuasa

**Affiliations:** 1 Department of Global Health Research, Graduate School of Medicine, Juntendo University, Tokyo 113-8421, Japan; 2 Faculty of International Liberal Arts, Juntendo University, Tokyo 113-8421, Japan

**Keywords:** purpose in life, spiritual health, older adults, rural Japan, psychosocial well-being

## Abstract

**Background**: As societies worldwide experience rapid aging, maintaining psychological well-being in later life is increasingly important. In Japan, where spirituality often takes noninstitutional forms, the concept of spiritual health remains underexplored. Purpose in Life (PIL), a core component of spiritual health, has been shown to predict well-being and healthy aging, yet its correlates remain unexplored in rural Japanese populations. This study aimed to assess PIL levels and examine its sociodemographic, social and health-related correlates among older adults living in Shimane Prefecture, a super-aged region in Japan. **Methods**: A cross-sectional survey (May–August 2024) included 308 adults aged ≥65 years. The 20-item PIL scale categorized respondents into low, moderate, and high PIL. Group differences were examined with chi-square/Fisher tests; unadjusted associations were used with ordered logistic regression. Multivariable analyses used a generalized (partial) proportional-odds model, adjusted for age and sex, with results reported as odds ratios and 95% CIs. **Results**: 42.9% of participants were classified as high PIL and 18.8% as low. Volunteering showed threshold-specific effects: it was unrelated to moderate PIL levels but was associated with approximately 2.4-times higher odds of high PIL (95% CI 1.32–4.38, *p* = 0.004). University education showed a trend-level association with higher PIL after controlling for false discovery rate (aOR 3.16, 95% CI 1.28–7.82, *p* = 0.013). The worse self-rated health was associated with lower PIL after adjustment. **Conclusions**: Subjective health and psychosocial engagement are key correlates of PIL in rural older adults. Culturally sensitive interventions may help promote purpose and well-being in aging populations.

## 1. Introduction

In recent years, life expectancy has increased significantly across the world, especially in developed countries, where aging populations are advancing rapidly due to declining birth rates (WHO, 2022). Traditional social systems have been structured around a linear life course, namely education, employment, and retirement. However, in today’s era of the ‘100-year life’, how people live meaningfully during the extended post-retirement phase has become a pressing issue (Yemiscigil et al., 2021). This issue is addressed in the book “LIFE SHIFT” by Gratton and Scott, which emphasizes the importance of purpose and reinvention in the context of extended lifespans (Gratton & Scott, 2016). As societies change in response, attention has increasingly turned to well-being, a concept that emphasizes not just length, but also quality of life (Musich et al., 2018).

In Japan, this shift is reflected in the international recognition of local practices and cultural concepts. For example, a specific region in Okinawa has been designated a ‘Blue Zone’ for longevity, and the Japanese term ikigai, which refers to a sense of purpose in life (PIL), is now used globally (Buettner & Skemp, 2016; García & Miralles, 2017). Recent longitudinal evidence from Japan indicates that ikigai is not only a cultural philosophy but also a measurable determinant of health: older adults with stronger ikigai show lower cardiovascular mortality (Miyazaki et al., 2022) and greater psychological and social well-being over time (Okuzono et al., 2022). Together, these findings reinforce the notion that ikigai—as a culturally grounded form of purpose—represents an essential component of subjective well-being in later life. These developments highlight a growing appreciation for the internal dimensions of human well-being, including personal meaning and purpose in life (Steger, 2012). In this context, the concept of spiritual health—defined as the ability to live with a sense of meaning and direction—has attracted increasing interest (Dhar et al., 2013). While Western countries have made significant progress in studying and applying spiritual health in fields such as medicine and community care, theoretical and empirical research in Japan and other parts of Asia remains limited (AshaRani, 2022).

However, it should be noted that Japan presents a unique cultural context in which spirituality is often perceived as ambiguous or associated with mysticism rather than organized religion (Kavanagh & Jong, 2019). Unlike many Western and Asian countries, where spiritual health has gained a greater social consensus, the concept remains underexplored and poorly integrated into public health discourse in Japan. In the late 1990s, the World Health Organisation (WHO) proposed the inclusion of spiritual health in its official definition of health (WHO, 1998). However, due to the abstentions of several countries, including Japan, the proposal was not adopted (Nagase, 2012). As a result, while spiritual health is internationally recognized as a vital dimension of well-being, it has not been thoroughly examined in the Japanese context.

Given Japan’s unique religious views and philosophical traditions, it is urgent to reconceptualize spiritual health in a culturally appropriate way for the Japanese population. Addressing this conceptual gap is essential to improve well-being in Japan’s rapidly aging society (Hibino et al., 2012). This study focusses on PIL as a key component of spiritual health. PIL is a psychological construct that reflects the degree to which individuals perceive their lives as meaningful and purposeful (Ryff, 1989). It is typically measured using a validated 20-item scale (Schulenberg & Melton, 2010). Recent international meta-analytic studies have shown that a higher sense of purpose in life is linked to both lower perceived stress (Sutin et al., 2024a) and a reduced risk of incident dementia (Sutin et al., 2023), highlighting purpose as a potential protective factor for both mental and cognitive health. Higher levels of PIL have been associated with fewer depressive symptoms, better cognitive and physical functioning, and healthier lifestyles (Musich et al., 2018), as well as reduced stress and lower all-cause mortality (Alimujiang et al., 2019). In particular, a 28-year prospective study has shown that people with sustained high levels of PIL exhibited significantly better cognitive health in older age (Sutin et al., 2024b). As such, PIL is considered a crucial psychological resource for promoting successful aging, particularly in super-aged societies such as Japan (Tanaka & Johnson, 2021). Recent systematic reviews have identified PIL as a core component of spiritual health, closely associated with psychological well-being, resilience, and participation in meaningful activities (AshaRani, 2022). Building on this foundation, our research team hypothesizes that ‘self-directed living’, the ability to choose and shape one’s own way of life, may serve as a culturally relevant dimension of spiritual health in Japan, where people often seek existential meaning outside institutional religious frameworks.

We situate PIL in later adulthood at the intersection of three domains: (i) resources and appraisal, (ii) social roles and capital, and (iii) human capital. First, we propose that purpose is sustained when individuals perceive sufficient material and health resources to pursue valued goals; in our data, this is proxied by financial satisfaction, household income/pension, self-rated health, and perceived health change. Second, purpose arises from active participation in meaningful social roles; we capture this with participation in social and volunteer activities. Third, education, when viewed through a life course lens, functions as a foundational resource that shapes access to meaningful roles and builds efficacy across adulthood. We tested this framework jointly in a rural Japanese setting (Shimane), where dense community networks and service constraints can increase the importance of social participation and perceived resources for maintaining PIL. Japan is one of the oldest societies in the world, with 29% of its total population aged 65 years or older as of 2023. In Shimane Prefecture, this proportion rises to 34.7%, and in Unnan City, our primary study areas, the aging rate reaches 41.2%. Most prior Japanese evidence on PIL comes from urban or mixed settings. Rural Shimane Prefecture differs in ways that plausibly change both the level and the determinants of PIL as follows: it has a super-aging, shrinking population with high old-age dependency; community life is coordinated through community volunteering that specifies roles and duties (for example, Osekkai conferences (Ohta et al., 2022)), and livelihoods are predominantly in agriculture and fisheries with seasonal work cycles and mutual-aid norms. These features imply that community participation and cohesion may contribute more to PIL than in cities, while health burdens and financial strain may exert stronger negative pressure due to fewer buffering services.

This study examines correlates of purpose in life (PIL) among older adults in rural Shimane, comparing the contributions of social participation, health appraisal, and economic/resource factors after accounting for sociodemographic characteristics. We hypothesized that social and volunteer participation, more favorable self-rated health, and higher financial satisfaction (beyond objective income or pension) would be positively associated with PIL, and we further anticipated a positive association between educational attainment and PIL.

## 2. Materials and Methods

### 2.1. Study Setting and Participants

This study used a cross-sectional design to examine associations between PIL and sociodemographic, social, and health-related variables among older adults living in the Unnan City community, Shimane Prefecture. Data were collected between May and August 2024. The recruitment was conducted through trusted community leaders and organizers of local social participation activities in Shimane Prefecture. Paper-based questionnaires were distributed at community centers, volunteer organizations, and local events such as “singing cafés,” which were held to foster social connection among older adults. Community leaders were previously informed about the inclusion criteria (65 years or older, live in the community, and capable of responding independently) and helped to invite suitable participants. Written informed consent was obtained from all respondents prior to completing the questionnaire.

A total of 390 responses were collected through this convenience-based, non-probability sampling approach. Because questionnaires were distributed during open community events without a defined sampling frame, a conventional response rate could not be calculated. After excluding 20 respondents under 65 years of age and 62 cases with missing data, 308 valid responses were retained for the final analysis. Given that missingness mainly involved eligibility and the outcome, a complete-case analysis (list-wise) was conducted.

### 2.2. Eligibility Criteria

Participants in this study were community-dwelling older adults aged 65 years and above who were able to provide informed consent. Individuals who had been diagnosed with dementia or cognitive impairment or who resided in long-term care facilities were excluded from participation.

### 2.3. Study Instruments

#### 2.3.1. Dependent Variable

The dependent variable in our study is PIL, which was developed by Crumbaugh and Maholick, based on Viktor Frankl’s logotherapy (Crumbaugh & Maholick, 1964). We used the officially published Japanese version of the PIL Test, originally authored by J.C. Crumbaugh and L.T. Maholick, based on the concept proposed by Viktor E. Frankl, and supervised for the Japanese edition by Tetsuo Okado (Professor, Seitoku University) and the PIL Research Group. This validated translation has been used in many Japanese studies as a validated instrument. The scale consists of 20 items, each of which is responded to in a 7-point Likert format, and all item scores are summed to yield total scores; higher values indicate a stronger sense of purpose. The total scores range from 20 to 140. Higher scores indicate a stronger sense of PIL. Following established classification criteria, scores ranging from 20 to 91 were categorized as low PIL, 92 to 112 as moderate PIL, and 113 to 140 as high PIL. The scale has already shown strong psychometric properties, including internal consistency (Cronbach’s α = 0.86–0.90), split-half reliability (0.77–0.85) and test–retest reliability (0.68–0.83) in previous studies (AshaRani, 2022). The Cronbach alpha value of this instrument in our survey data was 0.96.

#### 2.3.2. Independent Variables

Guided by the theoretical framework—resources and appraisal, social roles and capital, and human capital—we organized the independent variables as follows.

Resources and assessment: objective financial resources—household income (amount in yen) and pension receipt (yes/no); subjective resources—self-rated financial satisfaction (very satisfied, somewhat satisfied, somewhat dissatisfied, very dissatisfied); and health appraisal—self-rated health status (very healthy, fairly healthy, somewhat unhealthy, not healthy) and perceived change in health during the past year (better, no change, worse) (Idler & Benyamini, 1997). Although the survey included questions about current and past illnesses, these items were excluded from the analysis.

Social roles and capital: participation in social and volunteer activities (yes/no). Human capital: education level (primary or junior high/high school/junior college/university/other) and current employment status (employed/not employed). Covariates and other measures: age, gender (male/female), marital status (single/married/divorced/other), and living arrangement (living alone/cohabiting). Religiosity was assessed with a single item for religious belief (Strongly believe/Somewhat believe/Do not believe/Not sure). Participants who answered (1) or (2) were further asked to specify their faith(s) from the following list: Buddhism, Shintoism, Christianity, Catholicism, Taoism, folk beliefs, or others. This variable is treated as a brief proxy of religious orientation to limit the respondent burden in a community setting and does not capture multidimensional aspects of spirituality common in Japan (e.g., ancestor- or nature-based practices). The questionnaire was organized into separate sections, (i) sociodemographic, economic, social and health items (independent variables), and (ii) the Purpose in Life (PIL) scale (dependent variable), presented later with distinct instructions and response anchors. This procedural separation was used to reduce the potential bias of the common method.

### 2.4. Statistical Analysis

Because the study is cross-sectional, all estimates are interpreted as associations rather than causal effects. The results are described as higher or lower odds of PIL categories associated with covariates after adjustment. Descriptive statistics were used to summarize the characteristics of the participants. To explore differences between PIL groups (low, moderate, high), chi-square tests and Fisher’s exact tests were used for categorical variables, as appropriate. Univariate logistic regression analyses were performed to find unadjusted associations between PIL levels and individual variables. Variables found to be statistically significant in univariate analyses (*p* < 0.05), along with theoretically important covariates such as age, sex, and education, were included in multivariable logistic regression models. We summarize the fit of the model using McFadden’s pseudo-R^2^, AIC, and BIC; since AIC/BIC are comparative indices, interpretation emphasizes adjusted odds ratios and 95% CIs. Results are reported as odds ratios (ORs) with 95% confidence intervals (CIs). We computed E-values to evaluate the robustness of our key findings to potential selection bias. E-values for the point estimates and for the confidence interval limits closest to the null value were calculated. To illustrate practical significance, marginal predicted probability for the volunteering variable was estimated for a prototypical respondent (65–69-year-old woman, high school graduate with good self-rated health). The proportional odds assumption for the ordered logistic regression model was evaluated using the Brant test. A *p*-value < 0.05 was considered indicative of a violation of the assumption, and detailed results (χ^2^ and *p*-values for each variable) are presented in Supplementary Table S1.

All statistical analyses were performed using Stata versions 18 and 19 (StataCorp LLC, College Station, TX, USA), and a two-tailed *p*-value < 0.05 was considered statistically significant.

### 2.5. Ethical Considerations

This study was approved by the Ethics Committee of the Faculty of Medicine of Juntendo University (Approval Number: E23-0364-M01). Written informed consent was obtained from all participants prior to data collection. To ensure confidentiality, all responses were anonymized and securely stored in a password-protected offline system.

## 3. Results

### 3.1. Sociodemographic and Lifestyle Characteristics of the Participants

A total of 308 participants were included in the final analysis. The average age was 75.5 years (SD = 6.63), with a range of 65 to 96 years. 14.3% had completed only primary or junior high school, while 23.7% had a university or graduate degree. The majority of the participants were women (63.3%), currently married or partnered (71.1%), co-habiting (83.3%), receiving a pension (95.1%), currently unemployed (51.6%), involved in social (91.6%) or volunteer (56.8%) activities, as shown in Table 1. As expected, given the community-venue recruitment, 91.6% of participants reported social activity participation, indicating an over-representation of socially active older adults in our sample.

### 3.2. Association Between Purpose in Life Categories and Sociodemographic/Health Factors

We performed chi-square and Fisher’s exact tests to find differences between all variables according to PIL categories. PIL was found to be significantly associated with the variables: self-perceived financial situation, social activities, volunteer activities, self-rated health and perceived health change over the past year, as shown in Table 2.

### 3.3. Univariable Ordered Logistic Regression Analysis of Factors Associated with PIL

An ordered logistic regression analysis was conducted to examine the factors associated with higher PIL scores among the participants. In the first step, we performed a univariate analysis to find the effect size of the unadjusted association of each socio-demographic variable with PIL (Table 3). The analysis revealed that higher educational attainment was significantly associated with higher PIL. Compared to those with only primary education, participants with vocational or college education had 2.41 times higher odds of reporting higher PIL scores (95% CI 1.18–4.89, *p* = 0.015), and those with university or graduate level education had 3.14 times higher odds (95% CI 1.52–4.46, *p* = 0.002). Regarding financial status, participants who reported being ‘concerned’ about their current financial situation had significantly lower odds of higher PIL (OR = 0.41, 95% CI: 0.19 to 0.90, *p* = 0.026), and those who were ‘highly concerned’ had even lower odds (OR = 0.12, 95% CI: 0.03 to 0.46, *p* = 0.002), suggesting that financial insecurity can negatively impact one’s sense of purpose. Individuals who did not participate in social activities had significantly lower odds of high PIL (OR = 0.25, 95% CI: 0.11 to 0.57, *p* = 0.001), and those who did not participate in volunteer activities also had lower odds (OR = 0.50, 95% CI: 0.32 to 0.77, *p* = 0.002). Self-rated health also demonstrated strong associations. Compared to participants who rated their health as ‘good’, those who rated it as ‘poor’ had significantly lower odds of high PIL (OR = 0.18, 95% CI: 0.07–0.46, *p* < 0.001), while those who rated their health as ‘very poor’ had dramatically lower odds (OR = 0.01, 95% CI: 0.001–0.080, *p* < 0.001). Perceived health decline over the past year was linked to lower PIL. Participants who reported that their health was ‘worse’ compared to the previous year had significantly lower odds of high PIL (OR = 0.16, 95% CI 0.07–0.35, *p* < 0.001), and even those who answered ‘no change’ had lower odds (OR = 0.41, 95% CI 0.26–0.64, *p* < 0.001), as shown in Table 3.

**Table 4:** Multivariable generalized (partial) proportional-odds model.

Variable (Reference)	PO Status	Category	aOR at Cutpoint 1 (Medium-or-Higher vs. Low) [95% CI]	*p*-Value (Cutpoint 1)	aOR at Cutpoint 2 (High vs. Medium-or-Lower) [95% CI]	*p*-Value (Cutpoint 2)
Education (Primary or less)	Parallel	High school	1.81 [0.80–4.06]	0.152	1.81 [0.80–4.06]	0.152
		Vocational/Junior college	2.32 [0.93–5.82]	0.072	2.32 [0.93–5.82]	0.072
		University/Graduate	3.16 [1.28–7.82]	0.013	3.16 [1.28–7.82]	0.013
Current financial situation (No concern)	Non-parallel	Slightly concerned	1.74 [0.69–4.36]	0.239	0.59 [0.28–1.24]	0.165
		Concerned	0.63 [0.26–1.53]	0.309	0.63 [0.26–1.53]	0.309
		Highly concerned	0.38 [0.10–1.43]	0.152	0.38 [0.10–1.43]	0.152
Religious belief (None)	Non-parallel	Strong belief	2.85 [1.00–8.13]	0.051	2.85 [1.00–8.13]	0.051
		Moderate belief	1.43 [0.86–2.40]	0.171	1.43 [0.86–2.40]	0.171
		Don’t know	0.39 [0.10–1.49]	0.167	1.64 [0.41–6.64]	0.488
Social activity (Yes)	Non-parallel	No	0.17 [0.05–0.58]	0.005	1.12 [0.34–3.67]	0.850
Volunteer activity (Yes)	Non-parallel	No	1.20 [0.53–2.68]	0.662	0.42 [0.23–0.76]	0.004
Self-rated health (Good)	Parallel	Fair	0.68 [0.30–1.54]	0.358	0.68 [0.30–1.54]	0.358
		Poor	0.43 [0.14–1.30]	0.134	0.43 [0.14–1.30]	0.134
		Very poor	0.01 [<0.01–0.18]	0.001	<0.01 [<0.01–0.18]	<0.001
Perceived health change (Better)	Parallel	Neither yes nor no	0.54 [0.31–0.93]	0.026	0.54 [0.31–0.93]	0.026
		Worse	0.53 [0.16–1.78]	0.307	0.53 [0.16–1.78]	0.307

Note: PO status = proportional-odds status (Non-parallel variables have threshold-specific odds ratios; Parallel variables share the same OR across thresholds); aOR = Adjusted odds ratio; Model is adjusted for age and gender; Model parameters: Wald test of remaining parallel constraints χ^2^(13) = 17.79 (*p* = 0.166); pseudo-R^2^ = 0.152; AIC = 570.87; BIC = 670.32. The proportional-odds assumption was evaluated using the Brant test (global χ^2^ = 15.15, *p* = 0.034), which indicated partial violation, mainly for ‘no social activity’ and ‘no volunteer activity’ variables (Supplementary Table S2). Therefore, a generalized ordered logit model was adopted as the main specification. False-discovery-rate (FDR) correction was applied using the Benjamini–Hochberg procedure across 10 hypothesis tests (α = 0.05). The FDR-adjusted critical *p*-value for the second smallest test was 0.010; the education coefficient (*p* = 0.013) therefore became marginally significant.

### 3.4. Multivariable Generalized (Partial) Proportional-Odds Model of Factors Associated with PIL

In the multivariable analysis, we estimate a generalized (partial) proportional odds model (gologit2, autofit, robust SE) that includes education, current financial situation, social and volunteer activity, self-rated health and perceived health change, adjusted for age and gender (Table 4). Education remained positively associated with a higher PIL across thresholds: compared to primary or lower, the odds were higher for vocational/junior college (OR = 2.32, 95% CI 0.93–5.82, *p* = 0.072) and for university/graduate (OR = 3.16, 95% CI 1.28–7.82, *p* = 0.013); high school showed a smaller, non-significant association (OR = 1.81, 95% CI 0.80–4.06, *p* = 0.152). Volunteering showed threshold-specific effects: not volunteering (vs volunteering) was not associated with being in the moderate PIL group (cutpoint 1 OR = 1.20, 95% CI 0.53–2.68, *p* = 0.662) but was associated with lower odds of being in the high PIL category (cutpoint 2 OR = 0.42, 95% CI 0.23–0.76, *p* = 0.004). Predicted probabilities for a prototypical respondent computed for volunteering status indicated a 0.57 probability of being in the high Purpose in Life (PIL) category among volunteers and 0.37 among non-volunteers.

Social activity showed the opposite pattern: being not socially active (vs socially active) was associated with lower odds of being in the moderate PIL group (cutpoint 1 OR = 0.17, 95% CI 0.05–0.58, *p* = 0.005) but not at the upper threshold (cutpoint 2 OR = 1.12, 95% CI 0.34–3.67, *p* = 0.850). Self-rated health showed graded negative associations (e.g., very poor health had markedly lower odds of higher PIL), and perceived health “neither better nor worse” (vs better) was associated with lower PIL (OR = 0.54, 95% CI 0.31–0.93, *p* = 0.026); “worse” vs. better was not significant. Financial-concern categories and religious belief did not reach statistical significance after adjustment.

The E-values for the point estimates were 5.8 for university education, 4.2 for volunteering, and 3.1 for perceived health change. The corresponding E-values for the lower bounds of 95% confidence intervals were 1.9, 2.0, and 1.4, respectively. The results imply that it would take an unmeasured factor with a large effect to make the observed associations disappear, indicating that the findings are fairly robust to possible selection bias.

## 4. Discussion

The current study revealed that approximately 42% of participants had a high PIL and about 20% had a low PIL (Table 2). Consistent with our framework, educational attainment, self-rated health, volunteering, and financial satisfaction were associated with PIL (Table 4). Threshold-specific estimates indicated that volunteering related mainly to being in the high PIL group, whereas lack of social activity related to remaining in the low PIL group; other associations were directionally consistent with expectations. Prospective work in Japan and elsewhere likewise links purpose/ikigai and social participation to healthier aging trajectories, providing context for these cross-sectional associations. First, a clear association was observed between subjective financial satisfaction and PIL (Table 4). Not actual income but the feeling of being “financially secure” showed a stronger association with higher PIL, suggesting that perceived security and self-assessment may matter more than objective economic indicators (McKnight & Kashdan, 2009). Related studies also indicate that self-efficacy mediates the relationship between PIL and well-being, underscoring the role of perceived financial stability in shaping a sense of purpose (Czyzowska & Gurba, 2021).

Regarding health, self-rated health and perceived changes in health during the past year were strongly associated with PIL (Table 4). Older adults who perceived their health as good tended to have a clearer sense of purpose in life. Although this study did not include objective health indices, previous research has reported that PIL contributes to preventive health practices and physical health maintenance (Kim et al., 2014; Czekierda et al., 2017). However, as this was a cross-sectional study, the direction of causality should be interpreted with caution. Individuals with higher PIL may be more likely to engage in health-promoting behaviors and thus perceive better health, while volunteering and purpose may mutually reinforce each other through enhanced self-efficacy and social engagement. Consistent with this interpretation, one of the most notable findings of the present study was the robust association between PIL and subjective perceptions of health (Table 4). This aligns with theoretical frameworks suggesting that self-efficacy and positive cognition contribute to the development of PIL (Hooker et al., 2018). Interventions that engage in processes of self-awareness and meaning making—such as mindfulness, narrative therapy, and meaning-centered activities—are considered effective (Vos & Vitali, 2018). Enhancing PIL has been shown to have beneficial effects on physical health, cognitive function, and psychological resilience (Musich et al., 2018; Shin et al., 2022), making it a key component in improving the well-being of older adults in the community.

Participation in local and volunteer activities also showed significant associations with a higher PIL (Table 4). To illustrate the practical relevance of volunteering, marginal probabilities showed that a 65–69-year-old woman, high school graduate with good self-rated health had a 57% probability of high PIL if she volunteered, compared with 37% if she did not. This 20-percentage-point difference highlights the meaningful impact of volunteering on purpose in life among older adults Community involvement in education, culture, safety and mutual aid provides opportunities to develop social roles and a sense of contribution, serving as a foundation for experiencing meaning in life. In fact, retirement from volunteer activity has been associated with a lower PIL(Russell et al., 2023), underscoring the importance of sustained social participation in maintaining purpose in life.

In addition, attention should also be paid to the potential impact of social isolation and weak social networks on PIL among rural elderly populations. In rural areas, a decline in social connections has been linked to a loss of PIL (Hussain et al., 2023; Lee & Kim, 2024). Educational history was trend-level associated with higher levels of PIL in this study. This finding is consistent with previous research indicating that people with higher levels of education tend to report a stronger sense of PIL (Sumner, 2017). The authors suggest that education can improve purpose in life by promoting greater self-efficacy, future orientation, and opportunities for meaningful engagement. Especially in Japan, where academic background has historically been a strong determinant of social status and personal identity (Kariya, 2013), educational attainment among older generations may be closely related to feelings of self-worth and perceived social contribution.

However, these interpretations remain hypothetical; it is not necessarily education itself, but the psychological and social benefits derived from education, such as self-affirmation and enhanced social roles, that may influence PIL. More research is needed to clarify the underlying mechanisms linking educational attainment and PIL, particularly in cultural contexts such as Japan.

Our interpretations of associations are grounded in established theoretical frameworks. Drawing on Bandura’s Social Cognitive Theory (Bandura, 2013), we propose that favorable health appraisal, higher education, and financial satisfaction enhance self-efficacy, which facilitates the initiation and sustained pursuit of meaningful goals and thereby supports purpose in life (PIL). Experiences gained through social and volunteer roles can further strengthen self-efficacy, reinforcing purpose among engaged older adults. According to Self-Determination Theory (SDT) (Deci & Ryan, 2012), volunteering and higher education attainment can boost relatedness and competence, while financial satisfaction can bolster autonomy, plausibly linking them to sustained purpose in later life (Tang et al., 2020). Complementing these accounts is the Generativity theory by Erik Erikson (Ehlman & Ligon, 2012) which suggests that higher education broadens opportunities for generative action, while volunteering and social participation offer concrete channels to express generativity, together, strengthening purpose in life in older adults.

Regarding religious belief, although people with stronger faith showed a tendency toward a higher PIL in the univariate analysis, this association did not remain statistically significant in the multivariable model. This suggests that religious belief, at least as measured by self-reported intensity, may not serve as an independent correlate of purpose in life in the Japanese context. One possible explanation is that spirituality in Japan is often practiced outside formal religious institutions, with meaning derived from ancestral respect, seasonal rituals, and beliefs based on nature (Yoshizawa et al., 2024). This perspective is supported by a recent regional report showing that many East Asians, including Japanese respondents, report personal spiritual experiences (such as ancestral presence) and describe religious traditions more as “ways of life” than formal identities (Pew Research Center, 2024). These findings reinforce the notion that conventional categorizations of religiosity may not adequately reflect the cultural and existential dimensions of spirituality in East Asia. As such, binary or ordinal measures of ‘religious belief’ may be insufficient to capture the nuanced role of spirituality in purpose in life among older adults in Japan. Future research should consider measuring the quality of religious or spiritual engagement, such as the degree of voluntariness, personal meaning, or integration into daily life, rather than frequency or label alone.

Future, adequately powered studies should report marginal effects and, once available, relate changes in PIL to established, validated thresholds. They should also pre-specify and test gender- and age-based effect modification using interaction terms or stratified models. To test whether increasing structured volunteering raises purpose in life and downstream health, future research could leverage natural experiments (e.g., policy or program expansions that exogenously increase access to community activities) or pragmatic cluster-randomized trials at the municipality or community center level. Longitudinal and cross-cultural comparative designs can explore how PIL evolves and is influenced by cultural and social context, as recent findings suggest that PIL is not a static trait but can change across life domains and with aging (Sutin et al., 2024b; Pinquart, 2002). In parallel, municipalities in Japan have begun experimenting with empowerment-based community initiatives that bridge health promotion, social participation, and local governance. For example, Unnan City in Shimane Prefecture implemented the “*Feasibility Study for Building Healthy Communities through Public–Private Partnership*” with an annual budget of ¥43.96 million in FY 2023 (Policy Planning Division). This exploratory project—conducted in collaboration with CNC Inc. (formerly Community Nurse Company), an organization that trains and supports community nurses in social implementation and which also organized the “Osekkai Conferences” to promote mutual help and community engagement—aimed to develop sustainable networks for mutual support and empowerment-oriented health promotion (Unnan City, 2023). Such initiatives align with emerging international evidence showing that community nursing interventions can effectively improve health outcomes and strengthen social connectedness among residents (Yata et al., 2025). These initiatives are now expanding beyond Unnan City to other municipalities, including those in Nara and Hokkaido Prefectures, suggesting a growing movement toward community-driven, empowerment-based health promotion models in Japan. Although associations detected in this study were small in magnitude, small differences in PIL may still be relevant for well-being and community engagement, particularly in rural settings that are super-aging. There is no established clinical threshold or minimally important difference for PIL in older Japanese adults; therefore, we interpret the findings in terms of population well-being rather than clinical effect sizes. The wide confidence intervals reflect the sample size and convenience design.

This study has several strengths it focused on Shimane Prefecture, a region that experiences rapid aging, which provided practical insights into rural realities. In particular, community-led initiatives such as “Osekkai conferences” and resident-driven health activities in Unnan City deserve recognition as social environments that foster a sense of PIL (Ohta et al., 2022). Furthermore, by using the reliable 20-item version of the PIL scale and focusing on subjective health assessments and financial satisfaction, the study was able to shed light on aspects of well-being that are difficult to capture with conventional objective indicators.

However, this study has several limitations. Given the cross-sectional design, our findings are associational and should not be interpreted as causal. Second, participants were recruited mainly through community centers, volunteer organizations, and social events. As a result, the sample may have over-represented relatively active and socially engaged older adults, potentially under-representing those who are frail, isolated, or less involved in community activities. Indeed, 91.6% of participants reported social participation. Although 16% of missing data was removed by list-wise deletion, we did not perform multiple imputation. Hence, systematic attrition bias cannot be ruled out. These factors limit the generalizability of findings beyond similar community-engaged populations and to a broader range of older adults. Despite these limitations, this study offers valuable insights into how Purpose in Life relates to social and health factors among older adults in rural Japan.

## 5. Conclusions

This study explored the levels of PIL and its associated factors among older adults living in a rural region of Japan. The findings revealed that higher educational attainment, better self-rated health, and participation in volunteer activities were significantly associated with higher PIL. These results suggest that improving well-being in later life requires not only physical and economic support, but also attention to psychosocial and existential dimensions. In the Japanese context, where spirituality is often practiced outside formal religious institutions, fostering opportunities for meaning-making and interpersonal connection within the community may play a critical role. Future research should explore context-sensitive strategies and interventions that support perceived health and social roles, particularly in aging and depopulating regions.

## Figures and Tables

**Table 1:** Characteristics of Study Participants.

Variable	N = 308	Percent (%)
Gender		
Male	113	36.7
Female	195	63.3
Age (Mean ± SD)	75.5 ± 6.6	
65–69	66	21.4
70–74	70	22.7
75–79	96	31.2
80 and above	76	24.7
Education level		
Primary or Junior high school	44	14.3
High school	117	38.0
Junior college, technical college, vocational school	72	23.4
University and Graduate school	73	23.7
Other	2	0.7
Marital status		
Single	10	3.3
Married	219	71.1
Divorced	10	3.3
Widowed	67	21.8
Other	2	0.7
Living arrangement		
Living alone	51	16.6
With others	255	83.3
Employment status (a job that provides income)		
Yes	141	45.8
No	159	51.6
Self-reported financial situation		
No concern	45	14.6
Slightly concerned	199	64.6
Concerned	48	15.6
Highly concerned	11	3.6
Household income		
Less than 100,000 yen	12	3.9
100,000 yen to less than 200,000 yen	61	19.8
200,000 yen to less than 300,000 yen	77	25.0
300,000 yen to less than 400,000 yen	61	19.8
400,000 yen and more	70	22.7
Pension		
Yes	293	95.1
No	12	3.9
Faith or religious beliefs		
Strong	19	6.2
Moderate	129	41.9
None	144	46.8
I don’t know	13	4.2
Social activities		
Yes	282	91.6
No	24	7.8
Volunteer activities		
Yes	175	56.8
No	125	40.6
Self-rated health		
Good	27	8.8
Fair	227	73.7
Poor	44	14.3
Very poor	10	3.3
Perceived health change over past year		
Better	147	47.7
No change	133	43.2
Worse	27	8.8

**Table 2:** Socio-demographic characteristics and health perception across PIL categories.

Variable	Low PIL	Moderate PIL	High PIL	*p*-Value
Total	58 (18.8%)	118 (38.3%)	132 (42.9%)	
Gender				0.569
Male	22	39	52	
Female	36	79	80	
Age				0.251
65–69	12	30	24	
70–74	14	26	30	
75–80	14	31	51	
80+	18	31	27	
Education level				0.065
Primary or Junior high school	14	17	13	
High school	23	51	43	
Junior college, technical college, vocational school	10	28	34	
University and Graduate school	11	21	41	
Other	0	1	1	
Marital status				0.660
Single	2	4	4	
Married	37	86	96	
Divorced	1	3	6	
Widowed	17	25	25	
Other	1	0	1	
Living arrangement				0.873
Living alone	11	19	21	
With others	47	99	109	
Employment status				0.069
Yes	22	48	71	
No	31	69	59	
Self-reported financial situation				0.011
No concern	6	14	25	
Slightly concerned	31	82	86	
Concerned	13	18	17	
Highly concerned	6	3	2	
Household income				0.806
Less than 100,000 yen	4	4	4	
100,000 yen to less than 200,000 yen	13	24	24	
200,000 yen to less than 300,000 yen	11	32	34	
300,000 yen to less than 400,000 yen	12	25	24	
400,000 yen and more	13	23	34	
Pension				0.654
Yes	55	112	126	
No	1	5	6	
Faith or religious beliefs				0.180
Strong	2	8	9	
Moderate	17	50	62	
None	32	56	56	
I don’t know	5	3	5	
Social activities				<0.001
Yes	45	111	126	
No	12	6	6	
Volunteer activities				0.003
Yes	31	59	93	
No	27	59	39	
Self-rated health				<0.001
Good	1	9	17	
Fair	34	89	104	
Poor	14	19	11	
Very poor	9	1	0	
Perceived health change over past year				<0.001
Better	14	52	81	
Neither yes nor no	31	56	46	
Worse	12	10	5	

**Table 3:** Univariable Ordered Logistic Regression.

Variable (Reference)	Category	Odds Ratio (OR)	95% Confidence Interval	*p*-Value
Gender (Male)	Female	0.88	0.57–1.37	0.581
Age group (65–69)	70–74	1.15	0.62–2.15	0.657
	75–79	1.75	0.97–3.15	0.063
	80+	0.87	0.47–1.60	0.653
Education (Primary or less)	High school	1.60	0.83–3.05	0.157
	Vocational/Junior college	2.41	1.18–4.89	0.015
	University and Graduate school	3.14	1.52–6.46	0.002
Current financial situation (No concern)	Slightly concerneda	0.65	0.35–1.22	0.184
	Concerned	0.41	0.19–0.90	0.026
	Highly concerned	0.12	0.03–0.46	0.002
Household income (<100,000 yen)	100,000–199,999 yen	1.55	0.48–5.03	0.462
	200,000–299,999 yen	2.04	0.64–6.50	0.225
	300,000–399,999 yen	1.61	0.50–5.20	0.428
	≥400,000 yen	2.15	0.67–6.91	0.200
Religious belief (None)	Moderate belief	1.56	1.00–2.44	0.052
	Strong belief	1.60	0.66–3.90	0.302
	Don’t know	0.66	0.22–2.04	0.472
Social activity (Yes)	No	0.25	0.11–0.57	0.001
Volunteer activity (Yes)	No	0.50	0.32–0.77	0.002
Self-rated health (Good)	Fair	0.46	0.21–1.03	0.060
	Poor	0.18	0.07–0.46	<0.001
	Very poor	0.01	<0.01–0.08	<0.001
Perceived health change (Better)	Neither yes nor no	0.41	0.26–0.64	<0.001
	Worse	0.16	0.07–0.35	<0.001

Note: Unadjusted (bivariate) ordered-logit associations for comparability. Primary multivariable model: generalized (partial) proportional-odds (see Table 4). Univariable ORs are average cumulative effects across thresholds. Data on work status and presence of children were collected but excluded from the final model due to lack of significant association with Purpose in Life (PIL).

## Data Availability

The data supporting the findings of this study are not publicly available due to ethical and privacy considerations. Data may be available from the corresponding author upon reasonable request.

## Supplementary Materials

The following supporting information can be downloaded at: https://www.mdpi.com/article/10.3390/ejihpe15120250/s1, Table S1: Brant Test for the Proportional Odds Assumption (n = 308).

## Abbreviations

The following abbreviations are used in this manuscript:

PIL	Purpose in Life
