# Supplementary material for: Correlates of Purpose in Life and Their Potential Role in Successful Aging Among Older Adults in Rural Japan
Source: Eur J Investig Health Psychol Educ. 2025 Dec 9;15(12):250. doi: 10.3390/ejihpe15120250 (PMC12732226; doi:10.3390/ejihpe15120250)
Supplement: Supplementary file 1 [file ejihpe-15-00250-s001.zip › ejihpe-3890391-supplementary-done.pdf]

Appendix Table S1. Brant Test for the Proportional Odds Assumption (n = 308)

Variable	χ^2	p-value	Assumption violated?
**Education**	0.39	0.533	No
**Economic life satisfaction**	0.81	0.368	No
**Religious belief**	3.61	0.057	No (trend)
**No social activity**	7.04	**0.008**	**Yes**
**No volunteer activity**	6.00	**0.014**	**Yes**
**Self-rated health**	2.77	0.096	No
**Health comparison**	0.06	0.804	No
**Global test**	**15.15**	**0.034**	**Partially violated**

Note. *Brant test for proportional odds assumption after the ordered logistic regression (n = 308). The global test was significant (χ^2 = 15.15, p = 0.034), suggesting partial violation of the proportional odds assumption, primarily for "no social activity" and "no volunteer activity" variables ($p < 0.05$).*
A generalized ordered logit model was therefore estimated as a sensitivity check, yielding consistent directional associations.
